# F31. POLYGENIC RISK SCORES AND EARLY RISK ENDOPHENOTYPES IN CHILDREN AT GENETIC RISK OF SCHIZOPHRENIA AND BIPOLAR DISORDER: IMPLICATIONS FOR THE DEFINITION OF THE CHILDHOOD RISK STATUS

**DOI:** 10.1093/schbul/sby017.562

**Published:** 2018-04-01

**Authors:** Thomas Paccalet, Alexandre Bureau, Elsa Gilbert, Nicolas Berthelot, Pierre Marquet, Sébastien Boies, Valérie Jomphe, Daphné Lussier, Michel Maziade

**Affiliations:** 1 CIUSSS de la Capitale-Nationale - CERVO Brain Research Center; 2 Laval University; 3 Université Laval & CERVO Brain Research Centre; 4 Université du Québec à Trois-Rivières; 5 CERVO Brain Research Centre

## Abstract

**Background:**

Polygenic risk scores (PRS) of schizophrenia (SZ) or bipolar disorder (BD) are derived from genomewide association studies discriminating unrelated patients from controls. We have recently shown that both the SZ PRS and the BD PRS also distinguished affected patients from their non-affected adult relatives in a familial sample.1 Furthermore, the association of the SZ PRS with BD subjects and, reciprocally, of the BD PRS with SZ subjects support the shared susceptibility for these diseases.1 Importantly, new studies suggest that PRS would also distinguish the offspring at genetic risk from controls2 and may be associated with psychotic-like experiences and negative symptoms in adolescents of the general population.3 Little is known though about the contribution of the PRS in the risk prediction in children at genetic risk.

Our group and others have shown that the risk trajectory of high-risk children (HR) born to an affected parent can be characterized by their risk endophenotypes, i.e. specific cognitive deficits and psychotic-like or mood-like experiences in childhood that flag the neurodevelopmental origin of the illness. Children at risk accumulate these risk endophenotypes along their developmental trajectory and this aggregation is a predictor of later transition to illness.4,5

We hypothesized that since the PRS is a reflection of the genomic liability to illness, it would consequently relate to risk endophenotypes and their aggregation in children at risk. Our objectives were to evaluate i) the power of PRS to discriminate children at risk from healthy controls and, ii) the association of SZ and BP PRS to early risk endophenotypes in these children.

**Methods:**

The sample comprised 70 HR from the Eastern Quebec Kindred Study of multigenerational families densely affected by SZ and BD and 894 healthy controls from the CARTaGENE project. Whole genome SNP genotyping was performed from blood samples. Calculation of PRS was made according to our previous report.1 All HR were characterized using 4 established risk indicators4: cognitive impairments, psychotic-like experiences, childhood non-psychotic Axis 1 DSM diagnoses and episodes of poor functioning. Stratification of the HR by the presence of childhood trauma was also performed.

**Results:**

PRS distinguished HR from healthy controls (p<.05). Significant associations of SZ PRS and risk endophenotypes were detected for psychotic-like experiences (relative risk RR=1.4, p=.034) and, when stratifying for trauma, for the speed of processing cognitive domain (p=.049). Importantly, PRS was significantly higher in HR who aggregated psychotic-like experiences and axis 1 diagnoses (RR=3, p=.01), and a trend was detected with the aggregation of cognitive deficits, psychotic-like experiences and axis 1 diagnoses (p=.08).

**Discussion:**

PRS were associated with individual risk endophenotypes and with the aggregation of risk endophenotypes in children born to an affected parent. These results call for further study on the exact contribution to the childhood risk status of the genomic susceptibility indexed by PRS and the combination of risk endophenotypes. Considering that the clinically high-risk (CHR) status can be defined as a late phase of risk,6 the accumulation of risk indicators in childhood, including PRS and risk endophenotypes, document this early life period as the optimal timing for early intervention approaches.

**References:**

1. Boies et al., Am J Med Genet B Neuropsychiatr Genet, 2017

2. Fullerton et al., Am J Med Genet B Neuropsychiatr Genet, 2015

3. Jones et al., JAMA Psychiatry, 2016

4. Paccalet et al., Schizophr Res, 2016

5. Maziade, N Eng J Med, 2017

6. Seidman & Nordentoft, Schizophr Bull, 2015

